# Supplementary material for: PREventing Mild Idiopathic SCOliosis PROgression (PREMISCOPRO): A protocol for a randomized controlled trial comparing scoliosis-specific exercises with observation in mild idiopathic scoliosis
Source: PLoS One. 2023 May 8;18(5):e0285246. doi: 10.1371/journal.pone.0285246 (PMC10166530; doi:10.1371/journal.pone.0285246)
Supplement: S1 File — (DOCX) [file pone.0285246.s001.docx]

**Supplementary file 1. Specifications for interventional group**

In addition to receiving adequate self-mediated physical activity, a scoliosis specific exercise at least three days per week will be included in their prescription of self-mediated physical activity. Scoliosis specific exercises will be tailored individually to patients, focusing on patient education of active self-correction in 3 dimensional planes (3D), muscular stabilization of the corrected posture, and application to activities of daily living. The intervention will be delivered individually in 60-minute sessions, once every two weeks during the first 3 months and then every 6 months thereafter in conjunction to follow-ups. Additional single bolus outpatient sessions may occur when extra education is required to master the program. Patients will also be prescribed a self-mediated home exercise program to be performed in 30-minutes sessions at least three times per week.

Progression of intensity and degree of difficulty within the intervention is determined individually based on patients’ abilities to manage each module of the treatment. For example, a patient who quickly adapts active self-correction movements separately will faster progress to combine movements in all three planes than a patient having motor difficulties initially.

Motor learning can be described as a process associated with practice or experience leading to a behavioral change in the capability to produce a skilled action. The goals at the biomechanical and neuromotor levels are directed towards the patient’s learning of optimal correction of scoliosis curvature in 3D through muscle activation and postural movement strategies (self-correction). Furthermore, the goals at the bodily function and psychological levels are directed towards a behavioral implementation of self-correction strategies in activities of daily living (ADL’s) and the development of a positive body image. The core content of the intervention session is, stepwise, as follows:

*Learning active self-correction*

• Education of body posture and awareness of postural deficits on the 3 spatial planes by using visual (mirror) and tactile (contact in the various postures) and verbal (therapist) feedback.

- 1. • Learning active self-correction on the 3 spatial planes. Patients will have digital access to their individually tailored program and also direct contact with research personnel via Physitrack platform. Within the platform, patients can report difficulties, compliance and side-effects from intervention.
  2. • Training awareness of correction on the sagittal plane to ensure thoracic kyphosis and lumbar lordosis. The patient is given feedback from the therapist with the amount of corrective movement required in the sagittal plane to produce an apex of the lumbar lordosis at L2. This is trained in sitting and standing initially with tactile support and verbal cues for feedback. Assistance from parents will be applied initially with proper education from the responsible therapist, when the patient becomes more aware of the movement, it is then done with less feedback.
  3. • Training of the awareness of curve apex translation towards concavity on the frontal plane to attain more symmetrical vertebral column alignment.
  4. • Training to combine movements in the frontal and sagittal planes to attain cross-sectional derotation.
  5. • Learning trunk muscular stabilization strategies for self-corrective postures: Patients are instructed on how to recruit deep abdominal and paravertebral muscles through an abdominal hollowing technique to stabilize the trunk in corrected positions. In the case of thoracic curves, simultaneous maintenance of scapular girdle muscle tension and ventralization of the rib hump is used to reduce the convexity of thoracic curves and facilitate symmetrical thoracic breathing patterns during both inhalation and exhalation. Endurance of isometric contractions with loads that are one-third to two-thirds of maximal loads are trained in sitting and standing positions as well as during walking and activities of daily living.
  6. • Learning over-corrective side shift postural strategies to the opposite side of the primary curve in relaxed sitting and standing positions.
  7. • Patients are instructed to recognize and avoid scoliotic postures by implementing active self-correction and even relaxed over-correction side shift postural strategies as much as possible into daily activities.

*Implementation of active self-correction*

• Training trunk muscular stabilization and endurance in corrective postures during lower limb closed kinetic chain functional movements such as squats, forward lunges, sideways lunges and single leg standing. The difficulty of each exercise can be increased with growing balance demand in ergonomic lifting, walking, hopping and running situations. The patients are asked initially to perform these specific exercises with 10 repetitions x 3 sets included in their prescription of 60 minutes self-mediated exercise daily. When patients have mastered these exercises, they can instead focus on transferring these skills to similar activities of daily living or individual sporting and recreational activities of interest.

• Training trunk muscular stabilization and endurance in corrective postures during upper and lower limb closed kinetic chain functional movements. The difficulty of each exercise can be increased with growing neuromotor demand such during oculo-manual tasks and sport specific tasks. The patients are asked initially to perform these specific exercises with 10 repetitions x 3 sets included in their prescription of 60 minutes self-mediated exercise daily. When patients have mastered these exercises, they can instead focus on transferring these skills to similar activities of daily living or individual sporting and recreational activities of interest.
