# Supplementary material for: PREventing Mild Idiopathic SCOliosis PROgression (PREMISCOPRO): A protocol for a randomized controlled trial comparing scoliosis-specific exercises with observation in mild idiopathic scoliosis
Source: PLoS One. 2023 May 8;18(5):e0285246. doi: 10.1371/journal.pone.0285246 (PMC10166530; doi:10.1371/journal.pone.0285246)
Supplement: S2 File — (DOCX) [file pone.0285246.s002.docx]

**Supplementary file 2: Behavioural changes and increasing compliance**

The design of the study and provided interventions have arisen on a base where compliance and behavioural changes have an essential value. The COM-B framework has influenced the process in designing this clinical trial. The table below illustrates identified possible factors influencing compliance and changes in behaviour in terms of capability, opportunity and motivation.

| Capability | Opportunity | Motivation |
| --- | --- | --- |
| Regular sessions | Immediate access to experts | Education/information on disease |
| Instructions/illustrations | Mobile application | Parental support |
| Physical capabilities/exercise status | Free healthcare | Feedback from staff |
| Comorbidities/general health | Exercises not requiring equipment | Back pain |

*The behaviour change wheel*


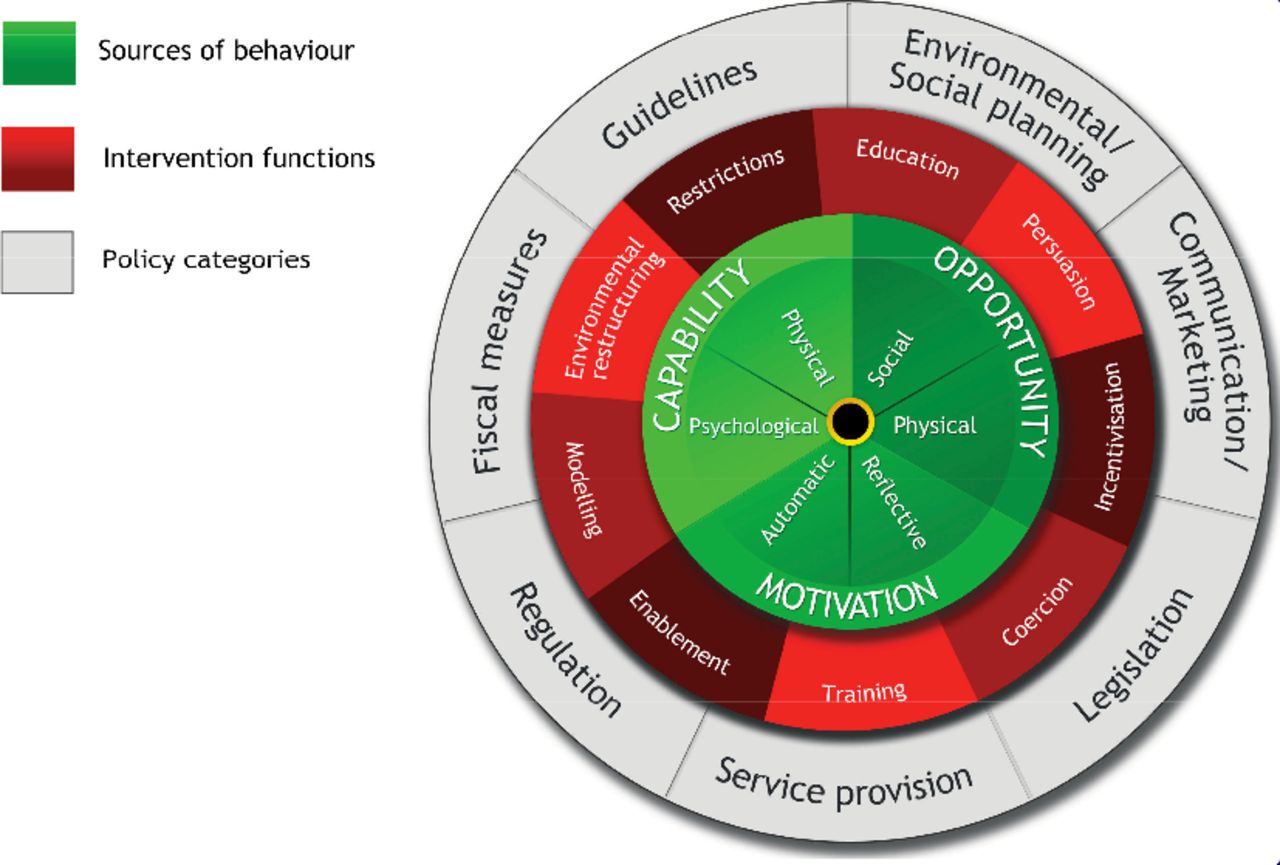


*Michie S et al. The behaviour change wheel: a new method for characterising and designing behaviour change interventions. Implement Sci. 2011;6:42.*
