# Supplementary material for: PREventing Mild Idiopathic SCOliosis PROgression (PREMISCOPRO): A protocol for a randomized controlled trial comparing scoliosis-specific exercises with observation in mild idiopathic scoliosis
Source: PLoS One. 2023 May 8;18(5):e0285246. doi: 10.1371/journal.pone.0285246 (PMC10166530; doi:10.1371/journal.pone.0285246)
Supplement: S3 File — (DOCX) [file pone.0285246.s003.docx]

**
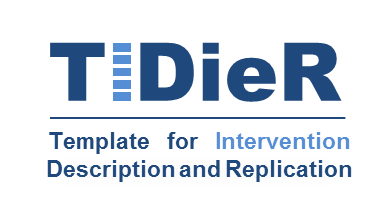
The TIDieR (Template for Intervention Description and Replication) Checklist*:**

Information to include when describing an intervention and the location of the information

| **Item number** | **Item** | **Where located **** | |
| --- | --- | --- | --- |
|  |  | Primary paper  (page or appendix  number) | Other ^†^ (details) |
|  | **BRIEF NAME** |  |  |
| **1.** | Provide the name or a phrase that describes the intervention. | SSE (Scoliosis-specific exercise) | ______________ |
|  | **WHY** |  |  |
| **2.** | Describe any rationale, theory, or goal of the elements essential to the intervention. | SSE has shown possible benefits in treating idiopathic scoliosis. There is however a lack of high-quality evidence. | _____________ |
|  | **WHAT** |  |  |
| **3.** | Materials: Describe any physical or informational materials used in the intervention, including those provided to participants or used in intervention delivery or in training of intervention providers. Provide information on where the materials can be accessed (e.g. online appendix, URL). | An individually tailored active exercise treatment strategy will be implemented to patients randomized to SSE group. Exercises will aim to achieve a correction in all three spatial planes. An online application will be used to monitor compliance and for patients to have access to their program (www.physitrack.com) | _____________ |
| **4.** | Procedures: Describe each of the procedures, activities, and/or processes used in the intervention, including any enabling or support activities. | All patients will have individual sessions every two weeks during the first 3 months to adopt the intervention. They will be instructed to carry out the exercises for at least 30 minutes per day and for at least 3 times a week. Additionally, patients will receive proper educational information in auto-correction in daily life and activities and correcting positioning in rest. When patients have adopted the intervention and can manage the exercises properly, general exercises will gradually be implemented with a correctional focus of the scoliosis. ____________ | _____________ |
|  | **WHO PROVIDED** |  |  |
| **5.** | For each category of intervention provider (e.g. psychologist, nursing assistant), describe their expertise, background and any specific training given. | Specialized physiotherapists with vast experience in managing and treating idiopathic scoliosis. All physiotherapists have had a rigorous training in the intervention provided prior to study start in order to assure standardized approach for all patients. Regular discussions and cooperation between the physiotherapists in terms of patient treatment and management will ensure standardized treatment approach. | _____________ |
|  | **HOW** |  |  |
| **6.** | Describe the modes of delivery (e.g. face-to-face or by some other mechanism, such as internet or telephone) of the intervention and whether it was provided individually or in a group. | For the first three months after inclusion, six individual face-to-face sessions will be offered to patients. Thereafter, by using the mobile application (Physitrack), patients have direct access to healthcare and research personnel for extra bolus sessions, inquires etc. The research personnel will be able to monitor pain levels, perceived intensity (RPE-scale) and compliance for every session the patient is having at home. | _____________ |
|  | **WHERE** |  |  |
| **7.** | Describe the type(s) of location(s) where the intervention occurred, including any necessary infrastructure or relevant features. | Location for the face-to-face sessions is in a university hospital setting. Thereafter, the intervention will mainly take place in the patients’ home environment. | _____________ |
|  | **WHEN and HOW MUCH** |  |  |
| **8.** | Describe the number of times the intervention was delivered and over what period of time including the number of sessions, their schedule, and their duration, intensity or dose. | Supervised sessions with physiotherapist six times for the first three months. Thereafter own sessions at home at least three days per week and for at least 30 minutes per day. Furthermore, educational aspects and implementation of auto-correction will be instructed to be performed daily. | _____________ |
|  | **TAILORING** |  |  |
| **9.** | If the intervention was planned to be personalised, titrated or adapted, then describe what, why, when, and how. | For each patient, exercises and interventions will be individually tailored based on clinical and radiological characteristics. Depending on each patient’s physical activity level and abilities, additional exercises concerning strength, coordination, proprioception and stamina may be added when the corrective exercises are being properly managed by the patient. All general exercises will be instructed together with the applied correction and consciousness regarding the individual curve. | _____________ |
|  | **MODIFICATIONS** |  |  |
| **10.^ǂ^** | If the intervention was modified during the course of the study, describe the changes (what, why, when, and how). | N/A | _____________ |
|  | **HOW WELL** |  |  |
| **11.** | Planned: If intervention adherence or fidelity was assessed, describe how and by whom, and if any strategies were used to maintain or improve fidelity, describe them. | All the physiotherapistsproviding interventions will have regular discussions and cooperation regarding the study participants together with the project manager. Standardized exercises for each curve type and for each spatial plane have been described and discussed with all clinicians, ensuring standardized procedures. In terms of addition of general exercises, each physiotherapist may decide for type of exercise based on patient characteristics. | _____________ |
| **12.^ǂ^** | Actual: If intervention adherence or fidelity was assessed, describe the extent to which the intervention was delivered as planned. | N/A | _____________ |

** **Authors** - use N/A if an item is not applicable for the intervention being described. **Reviewers** – use ‘?’ if information about the element is not reported/not sufficiently reported.

† If the information is not provided in the primary paper, give details of where this information is available. This may include locations such as a published protocol or other published papers (provide citation details) or a website (provide the URL).

ǂ If completing the TIDieR checklist for a protocol, these items are not relevant to the protocol and cannot be described until the study is complete.

* We strongly recommend using this checklist in conjunction with the TIDieR guide (see *BMJ* 2014;348:g1687) which contains an explanation and elaboration for each item.

* The focus of TIDieR is on reporting details of the intervention elements (and where relevant, comparison elements) of a study. Other elements and methodological features of studies are covered by other reporting statements and checklists and have not been duplicated as part of the TIDieR checklist. When a **randomised trial** is being reported, the TIDieR checklist should be used in conjunction with the CONSORT statement (see [www.consort-statement.org](http://www.consort-statement.org)) as an extension of **Item 5 of the CONSORT 2010 Statement.** When a **clinical trial** **protocol** is being reported, the TIDieR checklist should be used in conjunction with the SPIRIT statement as an extension of **Item 11 of the SPIRIT 2013 Statement** (see [www.spirit-statement.org](http://www.spirit-statement.org)). For alternate study designs, TIDieR can be used in conjunction with the appropriate checklist for that study design (see [www.equator-network.org](http://www.equator-network.org)).
