# Supplementary material for: PREventing Mild Idiopathic SCOliosis PROgression (PREMISCOPRO): A protocol for a randomized controlled trial comparing scoliosis-specific exercises with observation in mild idiopathic scoliosis
Source: PLoS One. 2023 May 8;18(5):e0285246. doi: 10.1371/journal.pone.0285246 (PMC10166530; doi:10.1371/journal.pone.0285246)
Supplement: S5 File — (DOCX) [file pone.0285246.s005.docx]

1. **Primary Registry and Trial Identifying Number**
   ClinicalTrials.gov, identifier: NCT05138393.
2. **Date of Registration in Primary Registry**
   12/01/2021
3. **Secondary Identifying Numbers**
   -
4. **Source(s) of Monetary or Material Support**
   All material will be funded by the research group.
5. **Primary Sponsor**
   Karolinska Institutet
6. **Secondary Sponsor(s)**
   -
7. **Contact for Public Queries**
   [elias.diarbakerli@sll.se](mailto:elias.diarbakerli@sll.se), tel: +460851770000, adress: Karolinska university hospital, 171 64, Stockholm, Sweden
8. **Contact for Scientific Queries**
   Elias Diarbakerli, PT, PhD. [elias.diarbakerli@sll.se](mailto:elias.diarbakerli@sll.se), tel: +460851770000, adress: Karolinska university hospital, 171 64, Stockholm, Sweden
   The contact for scientific queries must include:
9. **Public Title**
   Exercise treatment for Idiopathic Scoliosis
10. **Scientific Title**
    PREventing Mild Idiopathic SCOliosis PROgression (PREMISCOPRO):
11. **Countries of Recruitment**
    Sweden
12. **Health Condition(s) or Problem(s) Studied**
    Idiopathic scoliosis
13. **Intervention(s)**
    Experimental: Active self-corrective exercises

An active self-correction tailored to the individual type of curve and clinical presentation will be applied with the aim to correct the scoliosis in all three planes. Patients will also be informed and educated in task oriented activities of daily living. Training goals are directed towards postural control, spinal stability, muscular stabilization and endurance in corrective postures. Patients will have outpatient sessions once every two weeks the first 3 months and perform the exercises at home in 30-minutes sessions three times per week. Patients are encouraged to continue with non-specific self-mediated physical activities of moderate intensity at least 60 minutes daily. Compliance will be monitored through a mobile application (Physitrack) where the patients record their sessions and can have contact with the research personnel. A cognitive behavioral therapy approach to reinforce physical activity will be performed every 6 months.

Active Comparator: Observation

Patients are encouraged to continue with non-specific self-mediated physical activities of moderate intensity at least 60 minutes daily, for the entirety of the study. A cognitive behavioral therapy approach to reinforce physical activity will be performed every 6 months.

**Key Inclusion and Exclusion Criteria**
Inclusion Criteria:

- Cobb 15-24 degrees
- Skeletally immature, Sanders score of 4 or less and Risser less than 2.
- No menarche for females
- Aged 9-15 years
- No previous brace treatment or surgery for scoliosis
- Apex of the primary curve at T7 or caudal

Exclusion Criteria:

- Non-idiopathic scoliosis (i.e. neuromuscular, syndromic or congenital scoliosis)
- Previous spine surgery

1. **Study Type**
   Interventional study
   - Study design:
     - Randomized in an online module (www.swespine.se)
     - Single blinded (Outcome Assessor)
     - Single arm assignment
2. **Date of First Enrollment**
   First participant enrolled 9^th^ of June 2022.
3. **Sample Size**
   90 individuals planned to be enrolled.
4. **Recruitment Status**
   Recruitment status of this trial:
   - Recruiting: participants are currently being recruited and enrolled
5. **Primary Outcome(s)**
   Primary outcome measure is change in the Cobb angle of more than 6 degrees from baseline to the radiographic follow-ups and confirmed on two consecutive radiographs, similar to a previous randomized trial from our group
6. **Key Secondary Outcomes**
   Secondary outcome measures recorded at baseline and every six months for the entirety of the study include angle of trunk rotation, as measured with Bunnell’s scoliometer. patient-reported outcomes as measured with Scoliosis Research Society-22r, EQ-5D-youth version, Visual Analogue Scale-pain (VAS-pain), the International Physical Activity Questionnaire (IPAQ) short form and the pictorial part of Spinal Appearance Questionnaire (pSAQ) and hours in brace. Patients eventually also requiring brace treatment will be recorded.

At each follow-up additional questions regarding protocol fulfillment (own-perceived compliance of the treatment), patient satisfaction and adverse effects will be monitored.

1. **Ethics Review**
   Approved by the Swedish Ethical Review Authority (Diary number: 2020-06502).

Board Affiliation: Uppsala

Phone: +460104750800 Email: registrator@etikprovning.se

Address: BOX 2110, 75002, Uppsala, Sweden

1. **Completion date**
   Anticipated last patient finishing treatment: September 2027

Anticipated last patient follow-up: September 2037

**Summary Results**
-

1. **IPD sharing statement**

Plan to share IPD: Yes, IPD underlying published manuscripts will be accessible for other researchers. The investigators in charge will be responsible for reviewing access requests. Crude data, randomization procedures and intervention details can be shared with other researchers upon request.
