# Supplementary material for: PREventing Mild Idiopathic SCOliosis PROgression (PREMISCOPRO): A protocol for a randomized controlled trial comparing scoliosis-specific exercises with observation in mild idiopathic scoliosis
Source: PLoS One. 2023 May 8;18(5):e0285246. doi: 10.1371/journal.pone.0285246 (PMC10166530; doi:10.1371/journal.pone.0285246)
Supplement: Attachment — Submitted filename: Respond to reviewers.docx [file pone.0285246.s007.docx]

**Respond to reviewers**

*Academic editor*

Q: Criteria for included age, 10 years and 14 years to be revisited.

A: Thank you for the suggestion. The age span 9-15 years was chosen based on that adolescents, boys especially, may have a lot of remaining growth also after the age of 14. The lower threshold 9 was applied to be able to include children surpassing the age of 9 but not yet reached 10. The study is ongoing with a published protocol in clinicaltrials.gov. No changes have been made to the manuscript.

Q: Consider the measurement error of 5 degree while using Cobb's angle.

A: We have chosen 6 degrees in line with our previous publications (Diarbakerli et al, Plos one 2021 and Dufvenberg et al, J Clin Med 2021) to be able to make future comparisons between cohorts in these studies. This study is ongoing with a published protocol in clinicaltrials.gov. No changes have been made to the manuscript.

Q: Is sample size based on stratification adherence measured and compliance, not to be confused. To be made clear

A: We thank the editor for the question. We do not quite understand it completely but it is stated in the power calculation’s section that sample size is based on previous reported success rates. If the editor could elaborate on the question further we will be happy to answer it further.

*Reviewer 1*

Q: Intervention image with proper labelling can be added for more clearance about the technique.

A: We thank the reviewer for this suggestion. We have tried to describe the intervention in detail in the supplementary files. The core of the intervention is in line with previous literature and guidelines from SOSORT, namely that individual active correction in all planes is performed by the patients. Interventions can therefore differ for patients based on clinical characteristics and curve localization. No changes have been made to the manuscript.

Q: Reference manager should be followed for a citation and few of the references in the list are incomplete.

A: We thank the reviewer for these observations. We have updated the references accordingly.

*Reviewer 2*

Q: I would exclude patients below 10 years, so to include patients in a higher risk phase and only AIS. I would exclude also patients older than 14, since we can expect growth to be very close to the end. I understand that using the Sanders and Risser scores would help about this, but I would be more selective. Since curves up to 24° will be included, and the measurement error of Cobb is 5°, I would wait 30° to brace patients. Otherwise there is the theoretical risk to brace a patient that changes from 24 to 25 or 26 which is not actually a changed and showing and acceptable condition. I would also add other secondary outcomes: the number of improved patients (6° or more), the number of patients who completed growth below 30°, which is a main goal according to SOSORT guidelines. I think that reducing the progression rate even without totally stop it would be successful for the patients in case the threshold of 30° is not reached.
Please describe the protocol of exercises applied. Will it be SEAS, Schroth or other?
Blinding: I suggest to have a blind evaluation by an expert of the radiographies to doublecheck the treating physician's evaluation. This would make the paper much stronger.

A: We thank the reviewer for the questions and suggestions which we to a large extent agree upon. Regarding age, 9-15 years was chosen based on that adolescents, boys especially, may have a lot of remaining growth also after the age of 14. The lower threshold 9 was applied to be able to include children surpassing the age of 9 but not yet reached 10. These are also the threshold we have used in our previous studies.

In terms of brace initiation; it is stated in the manuscript that brace treatment will be offered to patients progressing > 6 degrees Cobb AND surpassing 25 degrees. A patient with a curve of 24 degrees with a follow-up radiograph showing a few degrees change will therefore not be equal to progression.

In terms of secondary outcomes, we have included a bunch both in terms of PROM data but also number progressing to the need of brace treatment which we believe is most interesting from a clinical perspective, that is, can we with SSE save more patients from ever needing a brace?

The methods used is a mix of the available literature and is mainly based on an active self-corrective strategy. As described in the manuscript and supplementary files, there is an exercise part (active self-correction) but also an educational part including activity in daily tasks where the child will learn how to move away from convexity to concavity, general loading of the spine, ergonomics etc.

When it comes to radiographs and evaluation of them, there will always be at least two persons assessing radiographs. If they can not reach consensus, a third expert will also be consulted. This is described in page 7 under the section outcome measurements. No changes have been made to the manuscript.

*Reviewer 3*

Q: Although the sample size is based on 90 people, did the authors consider stratification of perhaps for primary curve and age, as these were adjusted for in the models?

A: We thank the reviewer for the question. The power calculation is based on previous studies and success rates, with consideration for drop outs. No changes have been made to the manuscript.

Q: In the data analysis section, can the authors include that results will be present according to CONSORT.

A: We thank the reviewer for this suggestion. This has been added as the last sentence under the data analysis section, page 9.

Q: A CACE analysis will be used, can the authors define compliance here.

A: Information on compliance and recommended dose of exercises are described in page 5. Data on compliance will be extracted for the mobile application and also from surveys collected at each 6 month follow-up, detailed in pages 7-8.

Q: In the data analysis, it would help readability to split primary and secondary outcome analysis. Also some outcomes are collected at repeated time points, does the analysis take this into account?

A: The section data analysis discusses and describes how data will be handled. We believe the reviewer is referring to the section called outcome measurements where the primary and secondary outcome measurements are described in different sections. No changes have been made to the manuscript.

Q: Can the authors also state the results will be presented as treatment effect, confidence interval together with associated p-values.

A: We thank the reviewer for this suggestion. Since this is a study protocol, we have described how data will be handled thoroughly, including how for example categorical and continuous data will be presented. We refer to the data analysis section. No changes have been made to the manuscript.

Q: How is treatment adherence measured, not to be confused with compliance, or can the authors really clarify this

A: We thank the reviewer for this question. As stated in page 8, all study participants will answer three additional questions during each six-month follow-up; 1) the grade to which you feel that you have completed the treatment (adherence), 2) the grade to which you are motivated to carry out the treatment (motivation) and 3) how confident are you in your own capability to perform the treatment (capability). Additionally, the healthcare provider answers the question “to what grade the patient has adhered to treatment plan” (patient adherence). These questions are rated on a scale from best “very sure” (1 point) to worst “not at all” (4 points). No additional changes have been made to the manuscript.
