# Supplementary material for: PREventing Mild Idiopathic SCOliosis PROgression (PREMISCOPRO): A protocol for a randomized controlled trial comparing scoliosis-specific exercises with observation in mild idiopathic scoliosis
Source: PLoS One. 2023 May 8;18(5):e0285246. doi: 10.1371/journal.pone.0285246 (PMC10166530; doi:10.1371/journal.pone.0285246)
Supplement: Attachment — Submitted filename: Respond to reviewers2.docx [file pone.0285246.s008.docx]

**Respond to reviewers**

*Academic editor*

*Reviewer 1*

Q: All the queries raised are answered successfully by the authors. However, in the future studies more meticulously the limitations of the study should be addressed.

A: We thank the reviewer for the previous valuable comments and suggestions.

*Reviewer 2*

Q: Again, I think this is an overall fundamental and well designed study. Nevertheless, there are some points that are questionable since are not consistent with current literature.
Including patients younger than 10 means mixing JIS and AIS, makes results more confuse will take longer to reach the final outcomes increasing costs. Stating that this consistent with your previous publications is weak, since the rest of the world differs from this.

A: We thank the reviewer for this suggestion. As previously mentioned, the lower limit of age 9 years was applied in order to have the possibility to include patients being just below the age of 10 (i.e. 9 years and 11 months) and also to be in line with our previous studies in order to facilitate meta-analyses in the future. Since we have quiet strict criteria in terms of maturity (no menarche, Risser < 2 and Sanders 4 or below) we believe that many patients between ages 9-10 will be excluded if the lower border is 10 years strictly. It is true that some patients with JIS might be included but we believe they will be in clear minority and we do not believe this will affect the studies result.

Like previously mentioned; this is an ongoing study with a published protocol in clinicaltrials.gov. Changes in inclusion criteria is therefore not a possibility.

Q: There are 2 protocols with somehow solid data showing positive results in the field: Schroth and SEAS. To perform one of them the PTs need a specific certification. The authors state they will do something different coming from the literature and taking ideas from different approaches. What is the experience of the PTs administrating this treatment to patients? Do they have any certification (Schroth, SEAS or other)? How can readers know about the quality of the treatment? Please give more details.
Also, the protocol is not clear about how many training sessions will be administered after the initial phase. Again, Schroth and SEAS have a quite clear protocol to ensure quality and a continuous contact with the therapist, since if patients work too much by themselves the precision in the Active self correction tend to be less precise, and this can affect the final results.

A: We thank the reviewer for these important issues that are raised.

As we know, there is no level 1 evidence favoring scoliosis-specific exercises. This is not a generally accepted form of treatment for patients with idiopathic scoliosis. We do not intend to label the interventions as “Schroth”, “SEAS”, “FITS”, “side-shift therapy” etc and therefore a specific “certification” is not required for this study. SOSORT has stated in their latest guidelines (Negrini et al 2016) that the studies available are “generally of poor quality”. The guidelines state further:

*“SOSORT experts agree that PSSE should consist of the following:*

*Auto-correction in 3D*

*Training in activities of daily living (ADL)*

*Stabilizing the corrected posture*

*Patient education”*

All of these variables are addressed in the current study. Additionally, the study by Monticone et al from 2014 used a similar approach as this study protocol, not being tied to a specific “school” but to target the scoliosis patients individually based on above parameters agreed upon by SOSORT.

The senior physiotherapists involved in this study have long and vast experience in treating and managing scoliosis patients, both in terms of clinical and scientific point of view where the most senior PTs hold PhDs. The PTs have been participants and presenters in multiple SRS and SOSORT meetings. We have added the following sentence under the paragraph “interventions”: *The head physiotherapists have a long and vast experience in managing and treating idiopathic scoliosis patients, both from a clinical and scientific perspective (together approximately 20 years of experience).*

In the manuscript, it is stated on pages 5 and 6 (and supplementary files) under the headline “interventions” that patients in the interventional group will have outpatient sessions on 6 occasions during the first 3 months and thereafter will continue with their program according to prescription with follow-ups every six months. Above this, they have direct contact with research personnel via the mobile application described and are entitled to either digital or physical sessions whenever needed during the entire study period. In terms of frequency of exercises, SOSORT guidelines state “*The frequency of therapeutic sessions varies from twice to 7 days a week depending on the complexity of the techniques, motivation and the ability of the patient to carry out the treatment. Long-term outpatient physiotherapy sessions usually take place two to four times a week if the patient is willing to cooperate fully*”. Looking at the context for this study protocol, treatment is carried out at a large university hospital. The decision having outpatient sessions intense for the first three months is balanced against the willing and motivation for families to travel, leave school etc for a generally benign condition they are barely bothered by. Therefore, during the first three months, we aim to educate and make sure the child has a sense of control of his/her situation and treatment. If we identify difficulties, there are no restrictions for multiple outpatient sessions after the first three months. This is specified on page 5, last section.

Q: I appreciate the fact that radiographs will be assessed in blind. I would add a sentence about this also in the pragraph "blinding".

A: We thank the reviewer for this comment. It is stated in the manuscript that the statistician assessing outcome will be blinded for type of treatment. The following sentence have been added under the paragraph “blinding”: *In case of suspected progression during each follow-up including radiographs, two experts blinded for type of treatment will assess the radiographs to determine if progression has occurred.*

*Reviewer 3*

Q: All previous comments have been checked and the authors have addressed all comments. I have no further comments.

A: We thank the reviewer for the previous valuable comments and suggestions.
